# The Bacterial and Fungal Compositions in the Rhizosphere of *Asarum heterotropoides* Fr. Schmidt var. *mandshuricum* (Maxim.) Kitag. in a Typical Planting Region

**DOI:** 10.3390/microorganisms12040692

**Published:** 2024-03-29

**Authors:** Fuqi Wang, Zilu Zhao, Yangyang Han, Shiying Li, Xinhua Bi, Shumeng Ren, Yingni Pan, Dongmei Wang, Xiaoqiu Liu

**Affiliations:** 1 School of Traditional Chinese Materia Medica, Shenyang Pharmaceutical University, Shenyang 110016, China; fuqiwang2134@163.com (F.W.); zzilu98@163.com (Z.Z.);; 2 School of Pharmacy, Shenyang Pharmaceutical University, Shenyang 110016, China

**Keywords:** *Asarum*, microbial diversity, rhizosphere bacteria, rhizosphere fungi, network pattern

## Abstract

*Asarum* is a traditional Chinese medicinal plant, and its dried roots are commonly used as medicinal materials. Research into the traits of the bacteria and fungus in the *Asarum* rhizosphere and how they relate to the potency of medicinal plants is important. During four cropping years and collecting months, we used ITS rRNA gene amplicon and sequencing to assess the population, diversity, and predominant kinds of bacteria and fungus in the rhizosphere of *Asarum*. HPLC was used to determine the three bioactive ingredients, namely asarinin, aristolochic acid I, and volatile oil. The mainly secondary metabolites of *Asarum*, relationships between microbial communities, soil physicochemical parameters, and possible influences on microbial communities owing to various cropping years and collecting months were all statistically examined. The cropping years and collecting months affected the abundance and diversity of rhizosphere bacteria and fungi, but the cropping year had a significant impact on the structures and compositions of the bacterial communities. The rhizosphere microorganisms were influenced by both the soil physicochemical properties and enzyme activities. Additionally, this study revealed that *Trichoderma* was positively correlated with the three bioactive ingredients of *Asarum*, while *Tausonia* showed entirely opposite results. *Gibberella* and *Leptosphaeria* demonstrated a significantly negative correlation with asarinin and violate oil, but they were weakly correlated with the aristolochic acid I content. This study revealed variations in the *Asarum* rhizosphere microorganism population, diversity, and dominant types across four cropping years and collecting months. The relationship between *Asarum* secondary metabolites, the soil physicochemical properties, enzyme activities, and rhizosphere microorganisms was discussed. Our results will guide the exploration of the soil characteristics and rhizosphere microorganisms’ structures by regulating the microbial community to enhance *Asarum* quality.

## 1. Introduction

Rhizosphere microorganisms, which are the most active part of the soil ecosystem, are crucial for plant growth, nutrient circulation, and increasing yields [1,2]. Different microhabitats’ varieties and capacities for colonization through soil microbial communities influence the growth rates of pathogens and are crucial for enhancing plant health. Bacteria make up approximately 70–90% of the total number of rhizosphere microorganisms, which are sensitive indicators of nutrient changes in rhizosphere soil. They are the most numerous and extensively dispersed category of rhizosphere soil microorganisms [3]. When plants are infected, the abundance of bacteria and fungi will be altered. Potassium-solubilizing bacteria, phosphate-solubilizing bacteria (PSB), and nitrogen-fixing bacteria all showed lower relative abundances in rhizosphere soil, which may be related to an increase in saprophytic fungus [4,5]. Insoluble phosphorus can be transformed by PSB into usable phosphorus that plants can absorb and use later. According to the different substrates of PSB, they could be divided into organic phosphorus bacteria and inorganic phosphorus bacteria [6,7].

Numerous earlier research studies have demonstrated that the soil pH is the pivotal factor shaping the bacterial community in grasslands, farms, and forests [8]. In addition to soil bacteria, soil fungi also participate in the processes of nutrient cycling, and the distribution pattern of fungi has also been well researched. The quantity of phosphorus (P) that crops can absorb in the current growing season is known as the soil-accessible P content. In addition to promoting protein synthesis, P can improve plant disease resistance [9,10]. Inorganic nitrogen and relatively simple organic nitrogen that is readily decomposable in some organic compounds are included in soil alkali-hydrolyzable nitrogen (AN), also known as soil hydrolyzable nitrogen or soil-accessible nitrogen. The total amount of nitrogen that is easily hydrolyzed includes ammonium nitrogen, nitrate nitrogen, amino acid, amide, and protein nitrogen. Plant growth is directly correlated with the amount of AN present in soil [11,12]. Measuring the amount of AN in soil not only reflects the most recent nitrogen input but also provides a solid scientific foundation for fertilizing advice. The breakdown of organic matter and the cycling of nutrients in soil systems are two biochemical processes in which soil enzymes play a part. Soil enzymes are macromolecule active molecules with biocatalytic potential. Soil microorganisms, soil physicochemical characteristics, seasonal change, soil types, and plant variety all have an impact on soil enzyme activity. Therefore, understanding the distribution of microbial communities is crucial for enhancing ecological function and directing plant productivity, particularly for medicinal plants [13].

The region of the soil below ground that is impacted by roots is known as the rhizosphere, and it is distinct from the bulk soil around it in terms of its physicochemical qualities. It is surrounded by and affected by plant roots. Organic chemicals secreted by plants’ roots have the potential to change the community structure of the rhizosphere, which promotes microbial activity that helps plants absorb nutrients and defends them against pathogens [14]. Studies have demonstrated that rhizo-microbiomes are a component of the bulk soil community brought about by root selection. Reinhold-Hurek et al. [15] proposed a model in which microorganisms colonized from bulk soil to various root niches that showed that the microbial community composition of bulk soil differed significantly from that in the rhizosphere environment and that its diversity decreased with its proximity to the root. It has been established that rice, wheat, maize, and other crops all follow a similar microbial enrichment and colonization paradigm. It is yet uncertain, nevertheless, whether microbial enrichment and the colonization of medicinal plants from bulk soil to the rhizosphere adhere to the paradigm. In the soil around plants, the communication between the roots and the microbial populations of the soil is influenced by how near the soil is to the rhizosphere. The rhizosphere soil ecosystem is made up of plants and soil microbes [16]. In this system, soil and plants interact with microbes. The compositions of soil microorganisms can be influenced by plant root exudates and the physical and chemical characteristics of the soil, which, in turn, can have an impact on the physical and chemical characteristics of the soil and the development of plants. When growing and reproducing, soil microorganisms need to ingest particular soil components. In the course of their activities, they also produce a number of metabolites that affect the soil’s physicochemical composition and structure as well as the health and development of plants.

The possible interactions within typical ecological niche zones that community members share are investigated using a co-occurrence model analysis. It offers a fresh perspective on the composition and organization of microbial communities. The rhizosphere of wheat in bulk soil across China was found to be far more complicated than that of soybean according to network research. It was claimed that only specific microorganisms may colonize and thrive in the rhizosphere through the selection and filtering operations of roots. They also create certain metabolites throughout their activities, which have an impact on the physical and chemical compositions and structures of the soil as well as the plant’s quality or growth. Recent studies on the structure of the microbial community between the rhizosphere and bulk soil have frequently focused on agricultural and industrial crops [17]. However, little research has been conducted on the makeup of microbial communities and their co-occurrence patterns in areas connected to the rhizosphere of medicinal plants.

*Asarum*, also known as *Asarum sieboldii* Miq. var. *seoulense* Nakai or *Asarum sieboldii* Miq., is made from the dried roots and rhizomes of the plant *Aristolochia* chinensis. Its primary nutrients include volatile oils, lignans, flavonoids, and polysaccharides [18]. *Asarum* has a number of pharmacological effects, including analgesic, anti-inflammation, anti-oxidation, antibacterial, antitussive and anti-asthmatic, antidepressant, and anti-cancer effects, according to contemporary pharmacology and clinical investigations. Phlegm, cough, asthma, rheumatism, the wind and cold, and other types of discomfort are all treated with it. *Asarum* cultivation typically takes 4–5 years. However, in the long-term cultivation process, *Asarum*’s long-term absorption and consumption of soil nutrients, along with the release of root substances or decomposed stem and leaf components in the soil, will alter the physical and chemical properties of the soil, reduce its fertility, build up dangerous substances, alter the composition of the soil’s microbial community, and decrease the diversity of its microbial inhabitants [19]. In the end, it degrades *Asarum*’s quality and secondary metabolites. In order to understand the quality of *Asarum* from the perspective of soil microorganisms, it is crucial to understand the composition, distribution, and dynamic changes of the bacteria and fungus in the rhizosphere of the continuous crop *Asarum* [20,21].

In this work, soil samples from the rhizosphere of the priceless Chinese medicinal herb *Asarum* were gathered in Liaoning, China (one of the genuine producing areas), and a field experiment was carried out using a continuous cultivation strategy. The following goals guided our investigation. To start, we analyzed the diversity and predominant microbial community types in the soil samples from the *Asarum* rhizosphere. Second, we examined the impact of the harvest month and planting length on the rhizosphere microorganisms in monocultures. Thirdly, we investigated the connections between the secondary metabolites of *Asarum* and the soil’s microbial population, soil physicochemical characteristics, and soil enzyme activities. In order to increase the quality of *Asarum* by exploiting microbial resources, it is necessary to investigate the culture methods of healthy medicinal plants. This research will serve as a theoretical foundation and point of reference for the standardized, large-scale cultivation of *Asarum*.

## 2. Materials and Methods

### 2.1. Sample Gathering and Preparation

*Asarum* and its rhizosphere soil were collected from representative planting sites in the Baotang Village in the Xinbin Manzu Autonomous County of Fushun City (125°28′65″ E, 41°85′28″ N), Liaoning province of China. The *Asarum* plants at the first, second, third, and fourth cropping years were collected in October 2021 and June, July, and August 2022, respectively. Sampling was performed in the middle of each month. Before sampling, four replicate plots (10 m × 10 m each) were established, and a total of five sampling areas of 1 m × 1 m at the four corners and center of each plot were set. At each site, each plant sample was uprooted with a spade. After shaking the excess soil from the roots, approximately 1 mm of soil was kept attached to the roots. To ensure the uniformity of sampling, medicinal plants and their rhizosphere soil samples collected from different plots with different planting years and collection times were mixed in equal proportions and stored in 16 samples. The samples were put in plastic bags and immediately transported to the laboratory on ice. Part of the soil used for DNA extraction was stored at −80 °C. The soil used to determine the physicochemical properties was naturally air-dried. The roots of *Asarum* were naturally dried to determine the asarinin, aristolochic acid I (AAI), and volatile oil contents.

### 2.2. Determination of Bioactive Ingredients, Soil Physicochemical Properties, and Enzyme Activities

The contents of asarinin and AAI in *Asarum* were determined using HPLC with an external standard calibration curve method based on the Chinese Pharmacopoeia 2020 edition. Briefly, 0.5 g of dried roots (sieved at 0.25 mm) was dissolved in methyl alcohol and then sonicated and centrifuged to obtain the supernatant [22]. *Asarum* volatile oil was extracted by steam distillation. The air-dried and sieved (<2 mm) soil samples were used to determine the soil’s physicochemical properties, including pH, available phosphorus (AP), and alkaline nitrogen (AN). Soil pH was measured using a pH meter in a soil/water suspension (1:2.5 *w*/*v*) after shaking for 30 min [23,24]. The AN was determined using alkali diffusion methods. The AP was determined using the sodium bicarbonate extraction and molybdenum-antimony colorimetric method. Soil acid phosphatase activity (APT) was detected by the phenyl disodium phosphate colorimetric method. Soil sucrose convertase (INT) activity was determined by the 3, 5-dinitrosalicylic acid colorimetric method. Catalase activity (CAT) was assayed by potassium permanganate titration.

### 2.3. DNA Extraction and Sequencing

A 0.5 g portion of the rhizosphere soil sample was applied to extract DNA with the Fast DNA^®^ Spin Kit for Soil (MP Biomedicals, Solon, CA, USA) according to the manufacturer’s procedure. The DNA concentration and purity were detected and evaluated on a NanoDrop 2000 spectrophotometer (Thermo Scientific, Waltham, MA, USA) and by 1% (*w*/*v*) agarose gel electrophoresis. The 338F (5′-ACT-CCT-ACG-GGA-GGC-AGC-AG-3′) and 806R (5′-GGA-CTA-CHV-GGG-TWT-CTA-AT-3′) primers were used to amplify the V3-V4 fragment of the 16S rDNA gene. The fungal ITS1 fragment was amplified with the ITS1F (5′-CTT-GGT-CAT-TTA-GAG-GAA-GTA-A-3′) and ITS2R (5′-GCT-GCG-TTC-TTC-ATC-GAT-GC-3′) primers. Three technical replicates were established per sample. The PCR reaction is shown in the Supplementary Material.

The amplification products were checked on 2.0% agarose gel and purified using the AxyPrepDNA Gel Recovery Kit (AXYGEN, Union City, CA, USA) according to the manufacturer’s instructions. Afterward, a sequencing library was generated with the addition of an Illumina sequencing adaptor to the product using an Illumina TruSeq DNA Library Preparation Kit (San Diego, CA, USA) according to the manufacturer’s instructions. Finally, the library was sequenced on an Illumina MiSeq (PE300) platform at Shanghai Majorbio Bio-pharm Technology Co., Ltd. (Shanghai, China).

### 2.4. Microbial Community Analysis

Raw data were filtered for quality. The fastp (https://github.com/OpenGene/fastp, version 0.19.6, accessed on 21 December 2022) software was used for quality control and was carried out on the original sequencing sequence, and FLASH (https://ccb.jhu.edu/software/FLASH/index.shtml, version 1.2.11, accessed on 21 December 2022) software was used for Mosaic [25]. Abundance data of sequences matching “Chloroplast” and “Mitochondria” were removed from the data sets. The operational taxonomic units (OTUs) were clustered according to 97% similarity and assigned to each sample with the QIIME pipeline for sequence analysis [26]. The RDP classifier (https://sourceforge.net/projects/rdp-classifier/, version 2.13, accessed on 21 December 2022) with a 70% confidence threshold was used to assign taxonomic groups for the representative sequence in each OTU. The abundance information of the OTUs was normalized using a standard sequence number corresponding to the sample with the fewest sequences. All subsequent analyses were performed based on the normalized data.

### 2.5. Network Analysis

We performed the co-occurrence network analysis based on the analyzing tool, Networkx [27]. The top 200 bacterial and fungal OTUs for total abundance were selected. We set the *p*-value cut-off at 0.05 through the Spearman’s correlation coefficients > 0.7. Network visualization was performed on the Gephi platform. In each of these networks, the nodes that represent genus and edges represent significant co-occurrence relationships. Other topological properties of the network include the average degree, clustering coefficient, average path length, and betweenness centrality. The unstable edges were then filtered. The Gephi 0.10.1 software was used to explore network properties and visualize networks.

An ANOVA was performed to assess the differences in plant bioactive ingredients, soil physicochemical properties (pH, AP, and AN), and soil enzymes using SPSS 25.0 software (IBM, Inc., Armonk, NY, USA) among different groups.

### 2.6. Statistical Analysis

The alpha diversity was analyzed for rhizosphere bacteria and fungi at the OTU level using MOTHUR version 1.30.2 [28,29], and the OTU similarity level for index evaluation was 97%. Nonmetric multidimensional scaling analyses (NMDS) were carried out for microbial community data based on the Bray–Curtis distance. The distance matrices were calculated using QIIME, and the analysis and visualization were implemented with the “vegan” package. The analysis of similarities (ANOSIM) was applied with 999 permutations to examine the community composition difference between the compartments. Only the top 10 classes were analyzed, and the relative abundances < 0.01 were clustered into the ‘other’ group. Additionally, a Spearman’s rank correlation analysis was conducted to ascertain the correlations among the bacterial and fungal communities and environmental factors using the “pheatmap” package at the genus level. The top 30 genera were analyzed. Furthermore, a Linear Discriminant Effect Size (LEfSe) analysis was used to detect biomarkers that existed in different regions and growth stages using LEfSe (http://huttenhower.sph.harvard.edu/LEfSe, accessed on 21 December 2022). The non-parametric Kruskal–Wallis (KW) sum rank and the Wilcoxon rank-sum tests were used to determine the difference in species abundance between different groups, and then a linear discriminant analysis (LDA) was applied to estimate the influences of these different genera on the difference between the test groups. The LDA threshold was set as 2 and 4 for the rhizosphere microbe, respectively.

## 3. Results

### 3.1. Plant Bioactive Ingredients, Soil Physicochemical Properties, and Soil Enzyme Activities

From June to August in each year, asarinin accumulation increased; the content increased slowly from June to July, and increased significantly from July to August. There was a large value of asarinin in August in every cropping year. The content of AAI gradually accumulated from June to August each year and reached its peak in August. The third and fourth cropping years of *Asarum* collected in August exceeded the limit of (1.045 ± 0.006) × 10^−3^% and (1.32 ± 40.011) × 10^−3^%, respectively. The AAI content decreased rapidly from August to October each year. The content of volatile oil showed a slow cumulative increase trend year by year. The volatile oil content fluctuated significantly in the first and second cropping years, while the content fluctuated slowly in the third and fourth cropping years. The content of volatile oil was relatively low in July and October each year. The quality of *Asarum* harvested in July and August was qualified in this study. The fourth cropping year’s *Asarum* harvest at the end of July was best based on the growth law and economic advantages. At this time, *asarinin* and volatile oil were present in significant concentrations; meanwhile, the AAI stayed within acceptable limits (Table 1).

The soil physicochemical characteristics and soil enzyme activities are summarized in Table 2. The soil pH was significantly different in the sampling sites and ranged from 4.91 to 6.41, indicating that all soil samples were slightly acidic. The contents of AP, AN, and soil enzymes fluctuated with the harvest season and cropping years. The INT activity of *Asarum* with different cropping years was higher in June and lower in August. The INT activity of *Asarum* with the first and second cropping years was the highest in June, which was significantly different from that of the other months; this was probably because *Asarum* was in its vigorous growth period and needed sucrase to convert the sucrose in the soil into glucose and fructose for plant use to promote the growth of *Asarum*.

### 3.2. Microbial Composition and Diversity in Rhizosphere Soil

**Figure 1:**
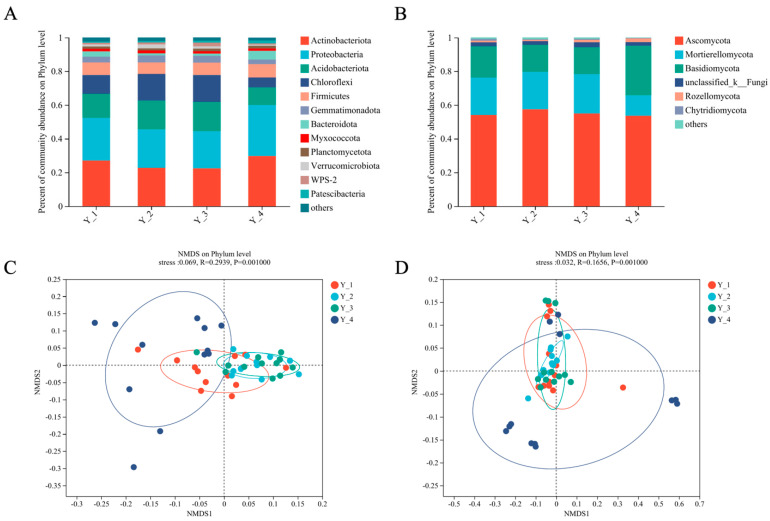
Relative abundance of dominant rhizosphere’s (**A**) bacterial and (**B**) fungal compositions grouped by cropping year at phylum classification level. Beta-diversity analysis of (**C**) bacterial and (**D**) fungal communities in different cropping years by a non-metric multidimensional scaling plot based on Bray–Curtis distance.

A total of 7483 bacterial OTUs and 3452 fungal OTUs were obtained from the 2,372,445 and 1,898,070 quality-filtered sequences, respectively. Bacterial phylum (*Actinobacteria*, *Proteobacteria*, *Acidobacteriota*, *Chloroflexi*, and *Firmicutes*) and fungal phylum (*Ascomycota*, *Mortierellomycota*, and *Basidiomycota*) were largely dominant in the two compartments (Figure 1A,B). Whether divided by cropping year or collection month, the species of bacteria and fungi in the *Asarum* rhizosphere soil at the phylum, class, and order levels were unchanged, but the abundance changed. As shown in Figure S1A (in the Supplementary Materials), there were slight fluctuations in the abundance of bacteria at the phylum level with different cropping years and collection months. As for the rhizosphere soil fungi, the abundance of *Ascomycota* and *Basidiomycota* was greatly affected by months (Figure S1B in Supplementary Materials). At the order level, presented in Figure S1C,E (in Supplementary Materials), *Micrococcales* were the dominant bacterial in the fourth year and in October. Figure S1D (in Supplementary Materials) demonstrates that the level of *Mortierellales* decreased significantly in the fourth year.

In this study, community diversity was reflected by the Shannon and Simpson index. Community richness was reflected by sobs (observed richness), the Chao1 estimator, and ACE estimator. The alpha diversity indices are shown in Table 3 and Table 4. The Kruskal–Wallis rank sum test that was performed on the data showed that the first three years’ bacterial alpha diversity was all significantly different from the fourth year, and some of the alpha indexes in other months were also significantly different from October (Table S1 in Supplementary Materials). Tables S2–S4 (in Supplementary Materials) demonstrate that some of the groups also showed significant differences in the fungal alpha diversity.

The results of the NMDS reflect the differentiation degree of the bacterial composition in the *Asarum* rhizosphere soil in different planting years and collection months (stress = 0.069). The difference in the bacterial community composition in the rhizosphere soil of Y_2 (the second cropping year of *Asarum*) and Y_3 (the third cropping year of *Asarum*) was small, followed by the difference in Y_1, and the difference in Y_4 was the largest (Figure 1C). Figure S2A (in Supplementary Materials) shows that there was a small difference in the bacterial community composition of the *Asarum* rhizosphere soil in August, followed by June, and there was a large difference in the composition structure of the bacterial community in October and July according to the collection time. The fungi NMDS results show the same trend as bacteria (Stress = 0.032). The results according to the growth time were more regular, with the community composition varying from Y_2 < Y_3 < Y_1 < Y_4 (Figure 1D), but there was no obvious change according to the collection months (Figure S2B in Supplementary Materials).

### 3.3. Effects of Cropping Year and Collecting Month on Rhizosphere Microbiome Compositions

**Figure 2:**
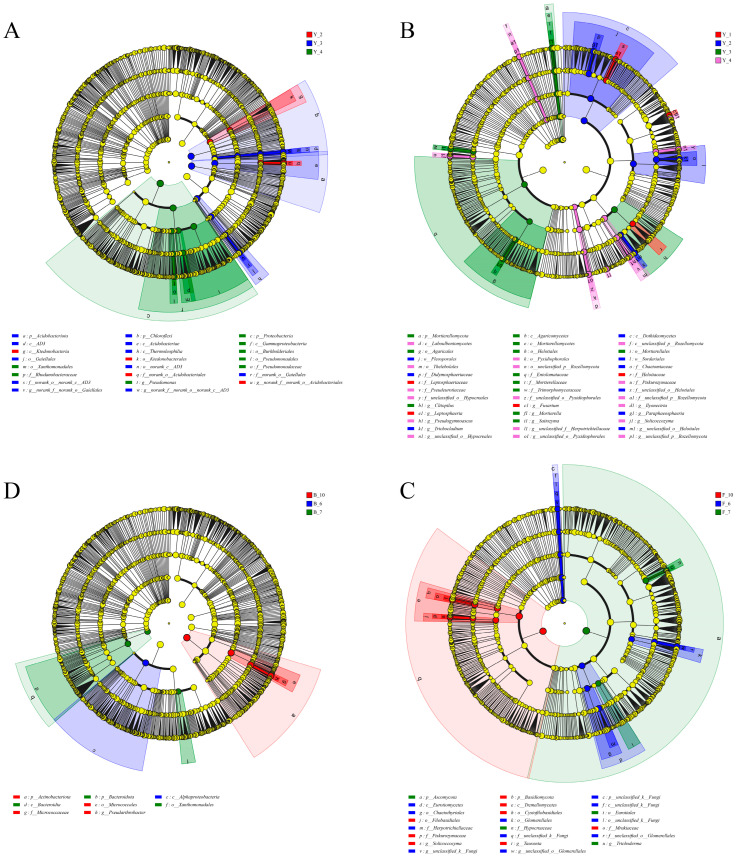
LEfSe diagram of rhizosphere (**A**) bacterial and (**B**) fungal communities grouped by cropping year. (**C**) Bacterial and (**D**) fungal communities were grouped by collecting month.

The LEfSe tool was used to pinpoint taxa whose relative abundances were differential among the groups that were tested. The differential features listed by LEfSe with an LDA score of 4 are shown in cladograms (Figure 2). As shown in Figure 2A, *Ktedonobacterales* (from class to order) was significantly enriched in the rhizosphere bacterial composition in Y_2; *AD3* (from class to genus) and *Gaiellales* (from order to genus) were significantly enriched in Y_3; and *Pseudomonas* (from phylum to genus), *Rhodanobacteraceae* (from phylum to family), and *Burkholderiales* (from phylum to order) were enriched in Y_4. *Acidobacteriales* was enriched in the second and the third cropping years. There was no species that was enriched in Y_1. *Pseudarthrobacter* (from phylum to genus) was enriched in October (Figure 2C).

There were more significantly different species in the fungal communities than in bacterial communities in the rhizosphere soil. *Asarum*’s significantly different fungal species in Y_1 only existed at the genus level, namely *Leptosphaeria*, *Fusarium*, and *Helotiaceae*; *Trichocladium* (from order to genus) and *Paraphaeosphaeria* (from class to genus) were enriched in Y_2, *Mortierella* (from phylum to genus) was mainly enriched in Y_3, in addition to *Clitopilus* (from class to genus), *Saitozyma* (from family to genus), etc. *Solicoccozyma* (from family to genus), *Rozellomycota* (from class to genus), *Pseudogymnoascus* (from order to genus), and *Pyxidiophorales* (from class to genus) were enriched in Y_4 (Figure 2B). *Tausonia* (from phylum to genus) and *Solicoccozyma* (from phylum to genus) were enriched in October. *Glomerellales* (from order to genus) and *Herpotrichiellaceae* (from class to family) were enriched in June (Figure 2D).

### 3.4. Environmental Factor Correlation Analysis

**Figure 3:**
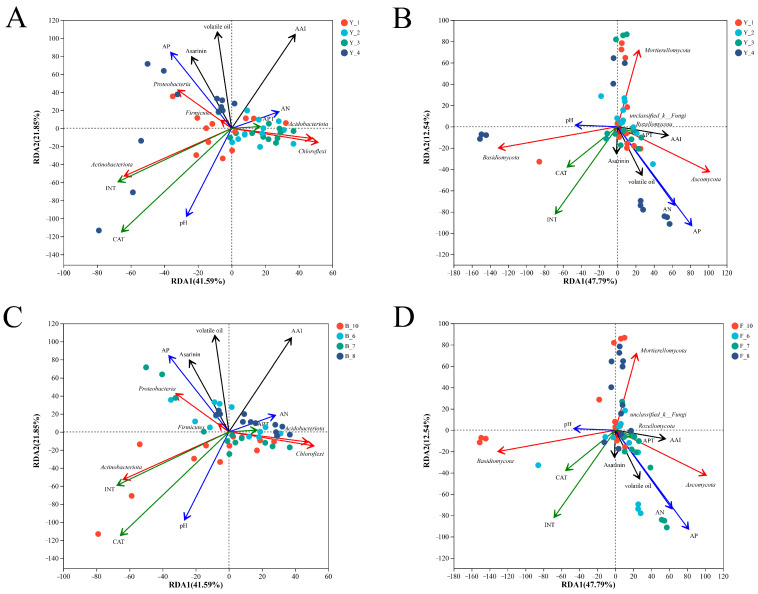
The ordination plots of the results from the redundancy analysis (RDA) to identify the relationships among the rhizosphere (**A**) bacterial and (**B**) fungal communities, three bioactive ingredients, the soil physicochemical properties, and the soil enzyme activities grouped by cropping year at the phylum level. The RDA analysis of the (**C**) bacterial and (**D**) fungal communities were grouped by collecting month.

The RDA analysis showed that the bacterial community in the rhizosphere of *Asarum* at the first cropping year was more dispersed, which may have been due to the abundance of soil bacterial community in the early growth stage (Figure 3A). *Acidobacteriota* and *Chloroflexi* were significantly correlated with the bacterial composition of the second and the third cropping years. *Chloroflexi* has a strong ability to degrade toxins. *Proteobacteria* was significantly correlated with the bacterial composition in the fourth year, and *Actinobacteriota* was closely related to INT and was significantly negatively correlated with the soil alkaline nitrogen content (Figure 3A). Some studies have shown that *Acidobacteriota* and *Actinobacteriota* are suitable for enrichment in nutrient-deficient environments. Asarinin, volatile oil, and AAI were significantly correlated with the samples collected in August with different cropping years, which may be related to the high content of bioactive ingredients in the samples collected in August (Figure 3C). There was no significant trend of rhizosphere fungi according to the cropping year (Figure 3B), but the influence of different months on fungi had certain rules. Specifically, *Ascomycota* was significantly associated with the fungal composition in June and July, and *Mortierellomycota* in August. *Basidiomycota* and *Mortierellomycota* were closely associated with the October samples (Figure 3D). From the above results, it can be inferred that different cropping years had a greater impact on the bacterial community differences, while the sample collection months had a greater impact on the fungal community differences.

### 3.5. Bacterial and Fungal Taxa Correlated with Asarum Bioactive Ingredients and Soil Physicochemical Properties

**Figure 4:**
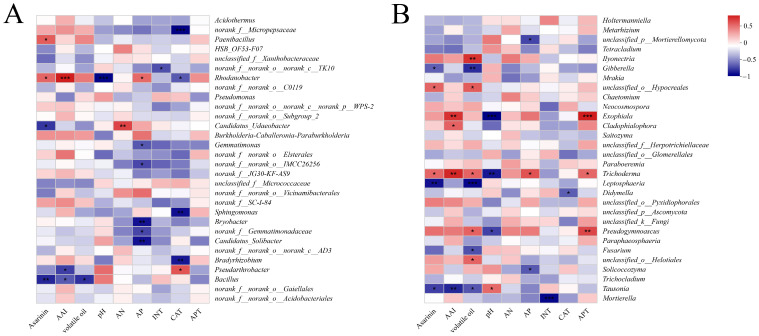
A correlation heatmap of the top 30 genera, three Asarum bioactive ingredients, and soil properties. The rhizosphere (**A**) bacterial and (**B**) fungal communities are shown, respectively. The correlation varies according to the right label color. * *p* < 0.05, ** *p* < 0.01, *** *p* < 0.001.

Spearman correlation heatmaps were used to investigate the correlation between the soil physicochemical characteristics and the rhizosphere bacteria and fungi of *Asarum* (*p* < 0.01). At the bacteria genus level, *Bacillus* was significantly negatively correlated with the content of asarinin. *Rhodanobacter* was positively correlated with AAI. *Bacillus* was negatively correlated with volatile oil. *Rhodanobacter* was negatively correlated with the soil pH value, and *Candidatus_Udaeobacter* was positively correlated with soil AN. *Bryobacter* and *Gemmatimonas* were substantially negatively correlated with the soil AP. *Bradyrhibium* and *Sphingomonas* had a negative correlation to CAT (Figure 4A).

At the fungal genus level, *Leptosphaeria* was significantly negatively correlated with asarinin. *Tausonia* was negatively correlated with AAI, while *Trichoderma* and *Exophiala* were positively correlated with AAI. *Leptosphaeria* and *Gibberella* were negatively correlated with the volatile oil content. *Ilyonectria* was positively correlated with the volatile oil content. *Trichoderma* and *Exophiala* were significantly negatively correlated with the soil pH. *Mortierella* was negatively correlated with the soil INT. *Pseudogymnoascus* and *Exophiala* were positively correlated with soil acid phosphatase (Figure 4B).

In general, there was a positive link between *Rhodanobacter*, *Trichoderma*, and bioactive ingredients and a negative correlation between *Bacillus*, *Tausonia*, and those same ingredients. *Gibberella* and *Leptosphaeria* were significantly negatively correlated with asarinin and volatile oil but weakly correlated with the AAI content. Therefore, we inferred that regulating the content of *Asarum* bioactive ingredients could be carried out by altering the ratio of key bacteria and fungi in the rhizosphere soil.

### 3.6. Co-Occurrence Pattern in Asarum Rhizosphere Soil

**Figure 5:**
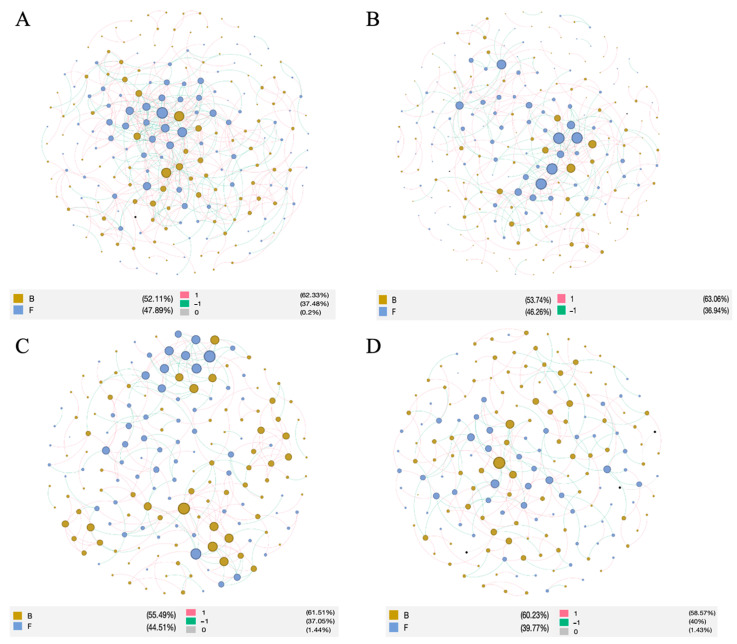
The network topology characteristics of the bacterial and fungal communities in the rhizosphere network. The edge is colored by the correlation relationship, and red and blue represent, respectively, the positive and negative correlations. The node is colored the main phylum. (**A**) The first cropping year, (**B**) the second cropping year, (**C**) the third cropping year, and (**D**) the fourth copping year.

The top 200 OTUs in each abundance were selected to construct the co-occurrence network of bacteria and fungi, and a total of four network maps were constructed. The network topology parameters showed that the microbial network in the rhizosphere soil had different structures according to the cropping year. The average geodesic distance, average clustering coefficient, average connectivity, and modularity of the molecular ecological network were larger than those of the random network, and the r^2^ values were all about 0.8, indicating that the co-occurrence network also met the characteristics of scale-free, small-world, and modular networks. It can be used in the subsequent study of microbial interrelations. The nodes and links were higher in Y_1 and Y_2 and lower in Y_3 and Y_4, and the modularity gradually increased, indicating that the modularity of the microbial community increased (Table 5). The number of positively correlated links was about 60%, and the number of negatively correlated links was about 40%. There was no significant change among the different cropping years, indicating that the proportion of competitive relationships between bacterial and fungal species was basically similar in this site (Figure 5). The network with one cropping year had the smallest average distance and the highest average connectivity and average clustering coefficient, indicating that the network structure of the co-occurrence of bacteria and fungi was unstable. They are more likely to be disturbed by the external environment. However, the Y_4 network showed the opposite trend, indicating that with the increase in cropping years, the structure of the co-occurrence network tended to be stable, and the plants were not susceptible to environmental disturbance.

Among the bacterial nodes, most of the nodal nodes were identified as *Proteobacteria* (range from 22.9% to 33.3%), *Actinobacteria* (range from 19.3% to 22.9%), *Acidobacteria* (range from 14.3% to 21.5%), and *Chloroflexi* (range from 4.7% to 18.8%). Among the fungal nodes, *Ascomycota* (range from 57.9% to 67.6%), *Mortierellomycota* (range from 8.69% to 15.2%), *Basidiomycota* (range from 8.8% to 17.4%), and *Rozellomycota* (range from 1.91% to 4.35%) were the main ones. The proportion of *Ascomycota* was the highest every year, and the proportion of *Rozellomycota* decreased with the increase in planting years. The characteristic *Glomomycota* hilum node appeared in the second and third years.

## 4. Discussion

Soil physicochemical properties are considered to be one of the key factors affecting the microbial composition. Root-associated microorganisms showed different changing trends under different planting years. *Actinobacteriota*, *Proteobacteria,* and *Acidobacteriota* were the most abundant bacterial groups in the rhizosphere. The relative abundances of *Actinobacteriota*, *Proteobacteria,* and *Acidobacteriota* varied under different cropping years, which was consistent with the previous results. *Ascomycota*, *Mortierellomycota,* and *Basidiomycota*, the dominant phylum in the rhizosphere, also showed fluctuating changes under different planting ages. Changes in the composition of the bacterial community may lead to variations in the metabolic capacity, bio-degradation, and disease-suppression abilities. Previous studies have shown that the compositions of bacteria and fungi in the rhizospheres of many plants, such as Arabidopsis [30], sugarcane [31,32], wheat [33,34], sugar beet [35,36], and soybean [37,38,39], can vary with the plant’s developmental stage. In actual production, the developmental stages of plants can generally be divided into four periods: seedling, development, flowering, and fruiting. It has been proven that different plant root exudates at different developmental stages lead to the formation of different rhizosphere microorganisms. As a perennial *Aristolochia* plant, it takes 4–5 years for *Asarum* to complete a growth period, so its growth and development stage will be related to the planting period. Some of the microorganisms with a function in biological control that have been substantially studied include *Actinobacteria*, which produce a wide variety of bioactive substances that prevent the growth of diseases in soil. Therefore, the cropping year is a key factor affecting the structure and composition of bacterial communities.

In this study, the community composition of bacteria and fungi in the *Asarum* rhizosphere was measured, but the bulk soil microorganisms and endo-rhizosphere microorganisms were not measured. It has been suggested that the alpha diversity would be higher in non-rhizosphere soils than in rhizosphere soils, which is possibly due to rhizosphere filtration and selection effects. Due to the strong screening and filtering effect of roots, the stability of the internal root community of medicinal plants can be ensured under different planting years [40]. Root deposition and root exudates affect the rhizosphere microbial composition [41]. However, due to the shielding effect of the root, only specific microorganisms can colonize the root. Because of this, the bacteria associated with the accumulation of active ingredients are more abundant in the rhizosphere than within the root. Therefore, it is of great importance to determine the microbial composition of the rhizosphere. However, non-rhizosphere soils are thought to be carbon-poor and therefore will be enriched for most oligotrophic bacteria. The rhizosphere environment recruits eutrophic bacteria due to root exudates. *Proteobacteria* are considered to be γ-strategic microorganisms that often live in nutrient-rich environments and tend to utilize unstable carbon sources [42]. The dynamics of *Acidobacteriota* were affected by the soil pH. *Actinobacteroa* could be used to reduce nursery fungal graft infections [43]. *Ascomycota* and *Basidiomycota* were also the dominant fungal phyla in the Astragalus rhizosphere.

The presence of microbe–plant symbiosis, which, in turn, influences microbial populations in roots and soil, is known to be influenced by the availability of soil N and P [44]. Plants recruit and choose phosphate-solubilizing bacteria for their roots in order to satisfy the P need. The organization of the microbial population in the rhizosphere also varies as a result of plant exudates. In order to boost P availability and contribute to P dissolution, plant roots can produce tiny molecular organic acids. This alters the makeup of bacterial microorganisms. Rhizosphere fungi have been linked to plant health and growth, plant residue breakdown, and nutrient delivery, and the makeup of the microbial population is correlated with the amounts of carbon, nitrogen, and phosphorus that are accessible in the soil.

The three bioactive chemicals identified in this study, namely asarinin, volatile oil, and AAI, are the primary bioactive compounds that contribute to *Asarum*’s pharmacological action and are also its secondary metabolites. The medicinal plants’ bioactive components evolve or build up over time. Additionally, temperature and precipitation can have significant impacts on how medicinal plants thrive. Secondary metabolites are often the building blocks on which medicinal plants act to produce their therapeutic effects. The environment and the medicinal plant itself during various growth stages frequently have a significant impact on the synthesis and accumulation of secondary metabolites [45,46]. Understanding the dynamics of secondary metabolites in medicinal plants throughout various growth years and seasons is therefore important for both the management of medicinal plant production as well as the collection and clinical use of traditional Chinese medicine. In order to make sure that the quality of *Asarum* fulfills the standards and that medicinal compounds are within a suitable range, we may establish the optimal harvest month and planting season for *Asarum* based on the results of Table 1.

In agricultural environments, soil microorganisms have a substantial impact on plant growth, nutrition, and health. An essential first step is to describe the richness and diversity of the microbial communities found in the healthy *Asarum*’s rhizosphere soil. There are certain issues with this planting technique despite the fact that the *Asarum* plants used for this study were healthy plants that were positioned continually in the same plot. Long-term continuous cropping has been linked to a number of issues, including an ecological imbalance of soil microorganisms, an enrichment of pathogenic microorganisms, and a decrease in beneficial microorganisms, according to reference [47]. This transition from bacterial to fungal dominance in soil microorganisms increases the likelihood that pathogenic bacteria may infect plants and result in a range of plant soil-borne illnesses. The absence of fungal dominant transformation traits in this study may have implications for the health of plant development. Other studies have demonstrated that the use of microbial organic fertilizer can balance out an out-of-balance flora, hence aiding in the prevention of soil-borne illnesses [48]. By examining the soil microorganisms and useful *Asarum* components, this work may offer some theoretical support and references for the use of microbial organic fertilizer in the production of medicinal plants.

## 5. Conclusions

We investigated the relationship between three secondary metabolites of *Asarum* and soil physicochemical parameters as well as the composition and diversity of the bacterial and fungal communities in the rhizosphere of *Asarum* grown continuously for four years. The *Asarum* rhizosphere’s bacterial and fungal compositions changed as the planting age and harvest month increased. Some particular floras have an impact on the buildup of *Asarum* metabolites. The *Asarum* rhizosphere’s bacterial and fungal populations changed as the cropping years and collection months increased. Asarinin, volatile oil, and AAI were negatively connected with *Bacillus* and *Tausonia*, and other dominating bacteria were also correlated with bioactive components. The rhizosphere bacteria *Rhodanobacter* and the fungus *Trichoderma* were favorably correlated with three bioactive ingredients. The main elements influencing the changes in the rhizosphere bacterial and fungal populations are the soil physicochemical characteristics and enzyme activity. We provided a theoretical foundation for using the microbial regulation of key flora to ensure the quality of *Asarum* and realize the standardized cultivation of medicinal plants by discussing the changes and effects of the continuous cultivation of *Asarum* on the soil microbial community and soil physicochemical properties.

## Figures and Tables

**Table 1:** The contents of Asarum’s three bioactive ingredients measured by HPLC.

No.	Asarinin (%)	Volatile Oil (mg/g)	AAI (% × 10^−3^)
10_1	0.109 ± 0.001 ^n^	1.67 ± 0.005 ^k^	0.163 ± 0.005 ^o^
10_2	0.396 ± 0.015l ^l^	2.22 ± 0.002 ^i^	0.174 ± 0.001 ^n^
10_3	0.523 ± 0.003 ^k^	2.50 ± 0.004 ^g^	0.251 ± 0.001 ^m^
10_4	0.856 ± 0.002 ^e^	2.63 ± 0.002 ^f^	0.386 ± 0.002 ^l^
6_1	0.283 ± 0.001 ^m^	2.47 ± 0.003 ^g^	0.587 ± 0.003 ^k^
6_2	0.520 ± 0.004 ^k^	2.94 ± 0.004 ^d^	0.611 ± 0.004 ^j^
6_3	0.539 ± 0.001 ^i^	2.87 ± 0.001 ^e^	0.612 ± 0.004 ^j^
6_4	0.940 ± 0.006 ^d^	3.15 ± 0.001 ^b^	0.669 ± 0.001 ^i^
7_1	0.284 ± 0.001 ^m^	2.06 ± 0.001 ^j^	0.775 ± 0.001 ^h^
7_2	0.528 ± 0.005 ^j^	2.31 ± 0.003 ^h^	0.816 ± 0.001 ^g^
7_3	0.618 ± 0.007 ^h^	3.09 ± 0.001 ^c^	0.865 ± 0.001 ^f^
7_4	1.129 ± 0.017 ^b^	3.33 ± 0.003 ^a^	0.932 ± 0.002 ^d^
8_1	0.668 ± 0.001 ^g^	2.50 ± 0.002 ^g^	0.896 ± 0.002 ^e^
8_2	0.704 ± 0.006 ^f^	2.50 ± 0.009 ^g^	0.956 ± 0.005 ^c^
8_3	1.108 ± 0.018 ^c^	2.86 ± 0.005 ^e^	1.045 ± 0.006 ^b^
8_4	1.303 ± 0.005 ^a^	3.31 ± 0.004 ^a^	1.324 ± 0.011 ^a^

Note: To simplify the description, “6_1” represents the sample of Asarum plants collected in June 2022 with the cropping age of 1 year. The values represent the means ± SD of the independent experiments. The values with superscript letters a–o are significantly different across each column (*p* < 0.05).

**Table 2:** The rhizosphere soil’s physicochemical properties and enzyme activities (n = 6).

No.	pH	AP (mg/kg)	AN (mg/kg)	CAT (mg/g)	INT (mg/g)	APT (mg/g)
10_1	6.41 ± 0.13 ^a^	29.09 ± 1.20 ^d^	76.46 ± 4.62 ^fg^	2.45 ± 0.02 ^c^	76.68 ± 2.16 ^d^	109.11 ± 13.17 ^ab^
10_2	6.32 ± 0.06 ^a^	19.03 ± 1.26 ^f^	96.76 ± 5.37 ^e^	2.16 ± 0.08 ^e^	52.93 ± 0.75 ^g^	104.16 ± 10.38 ^ab^
10_3	6.16 ± 0.12 ^b^	20.70 ± 3.48 ^ef^	98.17 ± 7.57 ^e^	2.25 ± 0.05 ^de^	55.76 ± 2.93 ^fg^	102.29 ± 2.16 ^ab^
10_4	5.81 ± 0.07 ^c^	17.82 ± 1.77 ^f^	76.76 ± 1.68 ^fg^	3.22 ± 0.04 ^a^	97.93 ± 2.21 ^a^	121.90 ± 17.33 ^ab^
6_1	5.82 ± 0.04 ^c^	36.13 ± 2.48 ^b^	92.52 ± 10.51 ^ef^	1.28 ± 0.02 ^g^	97.81 ± 1.81 ^a^	96.48 ± 8.44 ^b^
6_2	5.61 ± 0.04 ^de^	30.75 ± 1.25 ^d^	129.71 ± 6.73 ^bc^	2.14 ± 0.14 ^e^	76.11 ± 1.78 ^d^	121.22 ± 12.64 ^ab^
6_3	5.75 ± 0.04 ^cd^	24.09 ± 0.68 ^e^	101.45 ± 4.62 ^de^	2.37 ± 0.03 ^cd^	90.50 ± 5.05 ^bc^	108.29 ± 4.60 ^ab^
6_4	5.48 ± 0.08 ^ef^	32.50 ± 0.06 ^cd^	126.73 ± 0.84 ^bc^	1.20 ± 0.05 ^gh^	93.06 ± 4.73 ^ab^	118.57 ± 12.37 ^ab^
7_1	5.27 ± 0.07 ^gh^	45.62 ± 0.12 ^a^	133.87 ± 5.89 ^ab^	2.21 ± 0.06 ^e^	84.81 ± 3.05 ^c^	133.74 ± 15.92 ^ab^
7_2	5.42 ± 0.07 ^fg^	36.75 ± 0.74 ^b^	115.13 ± 15.56 ^cd^	2.99 ± 0.10 ^b^	75.61 ± 1.21 ^d^	152.20 ± 14.15 ^a^
7_3	5.40 ± 0.04 ^fg^	35.27 ± 1.47 ^bc^	146.07 ± 0.42 ^a^	2.38 ± 0.07 ^cd^	61.36 ± 2.90 ^f^	112.86 ± 21.54 ^ab^
7_4	5.18 ± 0.10 ^h^	43.27 ± 2.02 ^a^	108.29 ± 6.73 ^de^	2.46 ± 0.06 ^c^	56.55 ± 2.00 ^fg^	121.22 ± 23.88 ^ab^
8_1	4.87 ± 0.01 ^i^	19.73 ± 1.73 ^f^	77.15 ± 2.99 ^fg^	1.05 ± 0.01 ^i^	58.18 ± 2.86 ^fg^	127.19 ± 41.14 ^ab^
8_2	4.97 ± 0.04 ^i^	19.10 ± 1.67 ^f^	66.24 ± 5.78 ^g^	1.10 ± 0.07 ^hi^	56.30 ± 6.93 ^fg^	125.55 ± 33.55 ^ab^
8_3	5.41 ± 0.08 ^fg^	23.48 ± 2.36 ^e^	75.56 ± 2.52 ^g^	1.60 ± 0.06 ^f^	69.30 ± 6.16 ^e^	118.16 ± 27.15 ^ab^
8_4	4.91 ± 0.19 ^i^	32.39 ± 3.57 ^cd^	65.05 ± 13.11 ^g^	1.20 ± 0.01 ^g^	38.10 ± 2.36 ^h^	132.39 ± 36.13 ^ab^

Note: The values represent the means ± SD of the independent experiments. The values with superscript letters a–i are significantly different across each column (*p* < 0.05).

**Table 3:** The rhizosphere soil’s bacterial alpha diversity indexes for each soil sample.

No.	Shannon	Simpson	Sobs	ACE	Chao 1
10_1	6.18 ± 0.04 ^a^	0.009 ± 0.001 ^b^	2649 ± 60 ^abcd^	4329 ± 455 ^ab^	3870 ± 182 ^a^
10_2	6.34 ± 0.06 ^a^	0.006 ± 0.001 ^b^	2712 ± 55 ^abc^	3918 ± 85 ^ab^	3853 ± 92 ^a^
10_3	6.43 ± 0.03 ^a^	0.004 ± 0.000 ^b^	2727 ± 20 ^ab^	4174 ± 355 ^a^	3913 ± 96 ^a^
10_4	4.40 ± 0.49 ^c^	0.111 ± 0.047 ^a^	2107 ± 109 ^fg^	3706 ± 234 ^ab^	3182 ± 92 ^cde^
6_1	6.40 ± 0.08 ^a^	0.005 ± 0.001 ^b^	2689 ± 133 ^abc^	3747 ± 145 ^ab^	3727 ± 150 ^ab^
6_2	6.40 ± 0.01 ^a^	0.004 ± 0.000 ^b^	2668 ± 14 ^abc^	3816 ± 28 ^ab^	3795 ± 28 ^ab^
6_3	6.27 ± 0.06 ^a^	0.005 ± 0.001 ^b^	2497 ± 69 ^abcde^	4078 ± 244 ^ab^	3636 ± 121 ^ab^
6_4	6.38 ± 0.02 ^a^	0.004 ± 0.000 ^b^	2334 ± 38 ^ef^	3169 ± 68 ^b^	3142 ± 67 ^de^
7_1	6.33 ± 0.06 ^a^	0.004 ± 0.000 ^b^	2481 ± 111 ^bcde^	4233 ± 187 ^a^	3696 ± 191 ^ab^
7_2	6.20 ± 0.04 ^a^	0.005 ± 0.001 ^b^	2460 ± 53 ^bcde^	4200 ± 461 ^ab^	3623 ± 196 ^abc^
7_3	6.31 ± 0.06 ^a^	0.004 ± 0.000 ^b^	2444 ± 97 ^cde^	3926 ± 327 ^ab^	3591 ± 171 ^abc^
7_4	5.65 ± 0.21 ^b^	0.013 ± 0.002 ^b^	2001 ± 126 ^g^	3164 ± 212 ^b^	2918 ± 80 ^e^
8_1	6.42 ± 0.03 ^a^	0.004 ± 0.000 ^b^	2627 ± 24 ^abcd^	3962 ± 42 ^ab^	3722 ± 64 ^ab^
8_2	6.42 ± 0.01 ^a^	0.004 ± 0.000 ^b^	2643 ± 18 ^abcd^	4359 ± 381 ^a^	3855 ± 36 ^a^
8_3	6.25 ± 0.01 ^a^	0.005 ± 0.000 ^b^	2391 ± 47 ^de^	3703 ± 306 ^ab^	3434 ± 86 ^bcd^
8_4	6.44 ± 0.01 ^a^	0.004 ± 0.000 ^b^	2751 ± 25 ^a^	3976 ± 7 ^ab^	3957 ± 27 ^a^

Note: The values represent the means ± SD of the independent experiments. The values with superscript letters a–g are significantly different across each column (*p* < 0.05).

**Table 4:** The rhizosphere soil’s fungal alpha diversity indexes for each soil sample.

No.	Shannon	Simpson	Sobs	ACE	Chao 1
10_1	4.23 ± 0.01 ^ab^	0.042 ± 0.003 ^b^	724 ± 30 ^abcde^	966 ± 52 ^abc^	943 ± 51 ^abc^
10_2	3.82 ± 0.03 ^ab^	0.058 ± 0.002 ^b^	621 ± 2 ^def^	983 ± 87 ^abc^	868 ± 27 ^cd^
10_3	3.60 ± 0.04 ^abc^	0.074 ± 0.006 ^b^	541 ± 4 ^f^	790 ± 11 ^c^	731 ± 23 ^c^
10_4	1.84 ± 0.17 ^d^	0.370 ± 0.053 ^a^	289 ± 26 ^g^	619 ± 108 ^c^	481 ± 60 ^e^
6_1	3.49 ± 0.73 ^bc^	0.135 ± 0.111 ^b^	675 ± 135 ^bcdef^	1015 ± 59 ^ab^	918 ± 116 ^abc^
6_2	4.08 ± 0.05 ^ab^	0.049 ± 0.002 ^b^	770 ± 24 ^abcd^	1036 ± 56 ^ab^	1007 ± 30 ^abc^
6_3	4.49 ± 0.03 ^a^	0.030 ± 0.002 ^b^	787 ± 28 ^abc^	971 ± 26 ^abc^	968 ± 23 ^abc^
6_4	3.80 ± 0.04 ^ab^	0.066 ± 0.003 ^b^	644 ± 16 ^cdef^	900 ± 17 ^bc^	852 ± 36 ^cd^
7_1	4.29 ± 0.24 ^ab^	0.048 ± 0.018 ^b^	843 ± 27 ^a^	1220 ± 41 ^a^	1147 ± 39 ^a^
7_2	3.74 ± 0.53 ^ab^	0.105 ± 0.082 ^b^	699 ± 40 ^abcdef^	944 ± 35 ^abc^	939 ± 39 ^abc^
7_3	4.15 ± 0.03 ^ab^	0.039 ± 0.003 ^b^	705 ± 3 ^abcde^	936 ± 12 ^abc^	911 ± 10 ^abc^
7_4	2.78 ± 0.12 ^c^	0.121 ± 0.017 ^b^	298 ± 25 ^g^	640 ± 60 ^c^	485 ± 58 ^e^
8_1	3.98 ± 0.05 ^ab^	0.063 ± 0.003 ^b^	825 ± 31 ^ab^	1116 ± 52 ^a^	1100 ± 59 ^a^
8_2	4.02 ± 0.04 ^ab^	0.053 ± 0.003 ^b^	761 ± 9 ^abcd^	1096 ± 116 ^ab^	1017 ± 43 ^ab^
8_3	4.07 ± 0.03 ^ab^	0.043 ± 0.002 ^b^	653 ± 8 ^cdef^	919 ± 34 ^bc^	872 ± 30 ^bcd^
8_4	3.58 ± 0.09 ^abc^	0.096 ± 0.012 ^b^	583 ± 8 ^ef^	914 ± 127 ^bc^	846 ± 38 ^cd^

Note: The values represent the means ± SD of the independent experiments. The values with superscript letters a–g are significantly different across each column (*p* < 0.05).

**Table 5:** Parameters of molecular ecological network topological properties of bacterial and fungal communities in different cropping years.

Cropping Year		1	2	3	4
Molecular ecological networks	Similarity threshold	0.9	0.88	0.9	0.93
	Nodes	213	227	173	176
	Links	507	333	278	210
	Module	23	36	26	27
	Modularity	0.608	0.758	0.787	0.854
	Average geodesic distance	4.707	6.874	6.951	7.818
	Average clustering coefficient	0.265	0.227	0.279	0.197
	Average connectivity	4.761	2.934	3.214	2.386
	r^2^	0.871	0.844	0.791	0.865
Random networks	Modularity	0.420 ± 0.007	0.608 ± 0.010	0.561 ± 0.010	0.706 ± 0.010
	Average geodesic distance	3.453 ± 0.048	4.514 ± 0.104	4.232 ± 0.099	5.708 ± 0.214
	Average clustering coefficient	0.047 ± 0.008	0.017 ± 0.007	0.021 ± 0.009	0.009 ± 0.006

## Data Availability

Data are contained in the article.

## Supplementary Materials

The following supporting information can be downloaded at: https://www.mdpi.com/article/10.3390/microorganisms12040692/s1. Figure S1 Relative abundances of rhizosphere (A) bacterial and (B) fungal dominant phylum composition grouped by collecting month. Relative abundances of rhizosphere (C) bacterial and (D) fungal dominant order composition grouped by cropping year. Relative abundances of rhizosphere (E) bacterial and (F) fungal dominant order composition grouped by collecting month. Figure S2 Beta-diversity analysis of (A) bacterial and (B) fungal community grouped by collecting month by a non-metric multidimensional scaling plot based on Bray-Curtis distance. Table S1 Alpha diversity index statistics of bacterial grouped by cropping year. Table S2 Alpha diversity index statistics of bacterial grouped by sample month. Table S3 Alpha diversity index statistics of fungal grouped by cropping year. Table S4 Alpha diversity index statistics of fungal grouped by sample month.

## Abbreviations

AAI, aristolochic acid I; AN, Alkali hydrolyzed nitrogen; AP, available phosphorus; APT, acid phosphatase; CAT, catalase; INT, sucrose convertase; OTU, operational taxonomic units.
