# Supplementary material for: The Bacterial and Fungal Compositions in the Rhizosphere of Asarum heterotropoides Fr. Schmidt var. mandshuricum (Maxim.) Kitag. in a Typical Planting Region
Source: Microorganisms. 2024 Mar 29;12(4):692. doi: 10.3390/microorganisms12040692 (PMC11051765; doi:10.3390/microorganisms12040692)
Supplement: Supplementary file 1 [file microorganisms-12-00692-s001.zip › microorganisms-2918227-supplementary.pdf]

Supplementary material

PCR amplification of soil bacterial: Each sample was amplified by PCR using a 20 μL reaction with 5 M of each primer. The PCR system included 5×bufer (4 μL), 2.5 mM dNTPs (2 μL), 5 μM forward and reverse Primer (0.8 μL), 2.5 U/μL FastPfu Polymerase (0.4 μL), 20 mg/mL BSA (0.2 μL) and Template DNA (10 ng), adding ddH_2O to 20 μL. The PCR reactions were performed by an ABI GeneAmp® PCR System 9700 (Thermo Fisher Scientific) that used the following cycling conditions: initial denaturation step at 95°C for 3 min (1 cycle); 27 cycles at 95°C for 30 s, 55 °C for 30 s, 72°C for 45 s, a final extension step at 72°C for 10 min (1 cycle).

PCR amplification of soil fungi: Each sample was amplified by PCR using a 20 μL reaction with 5 M of each primer. The PCR system included 10×bufer (2 μL), 2.5 mM dNTPs (2 μL), 5 μM forward and reverse Primer (0.8 μL), 2.5 U/μL rTaq Polymerase (0.2 μL), 20 mg/mL BSA (0.2 μL) and Template DNA (10 ng), adding ddH_2O to 20 μL. The PCR reactions were performed by an ABI GeneAmp® PCR System 9700 (Thermo Fisher Scientific) that used the following cycling conditions: initial denaturation step at 95 °C for 3 min (1 cycle); 35 cycles at 95°C for 30 s, 55°C for 30 s, 72°C for 45 s, a final extension step at 72°C for 10 min (1 cycle).

Figure S1. Relative abundances of rhizosphere (A) bacterial and (B) fungal dominant phylum composition grouped by collecting month. Relative abundances of rhizosphere (C) bacterial and (D) fungal dominant order composition grouped by cropping year. Relative abundances of rhizosphere (E) bacterial and (F) fungal dominant order composition grouped by collecting month.

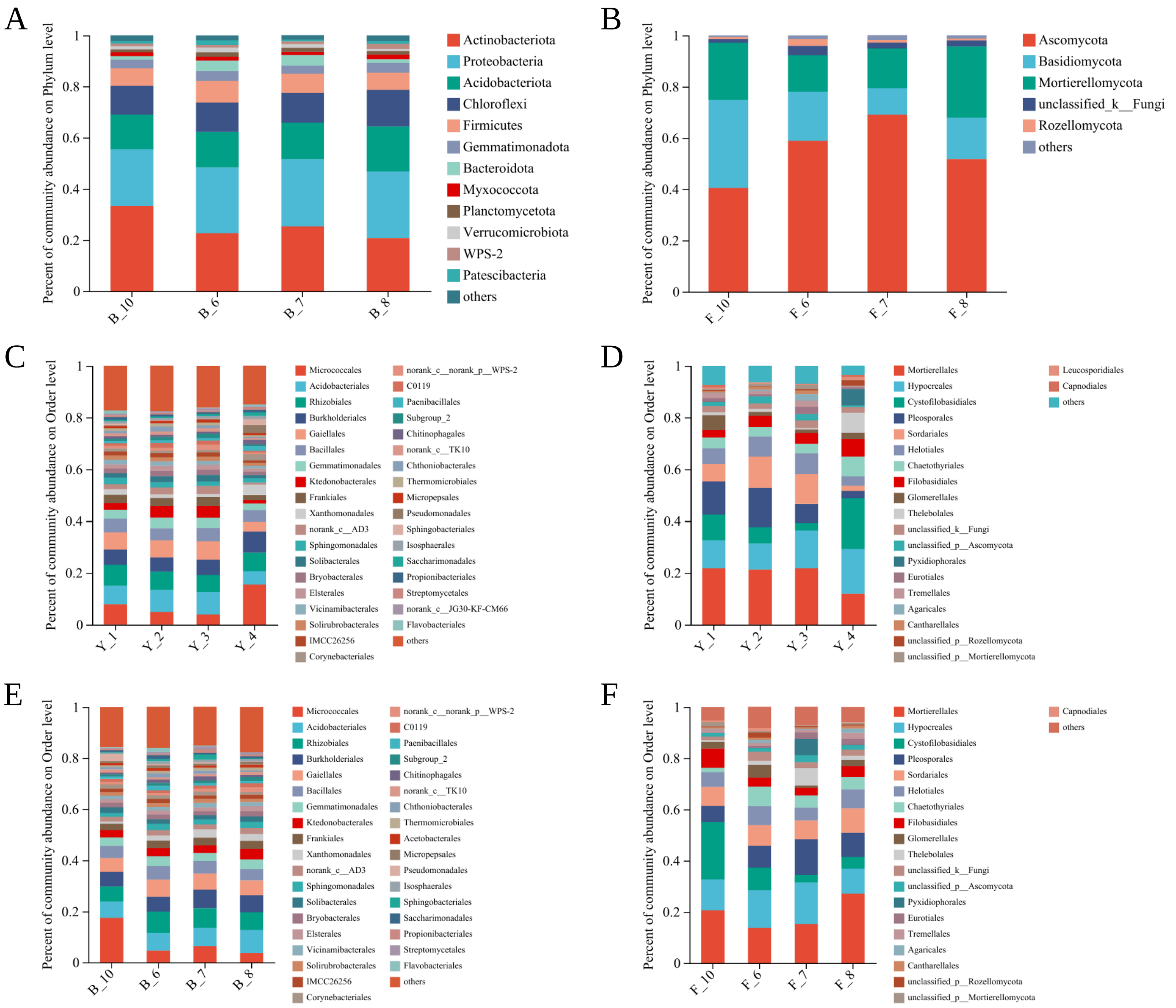

Figure S2. Beta-diversity analysis of (A) bacterial and (B) fungal community grouped by collecting month by a non-metric multidimensional scaling plot based on Bray-Curtis distance.

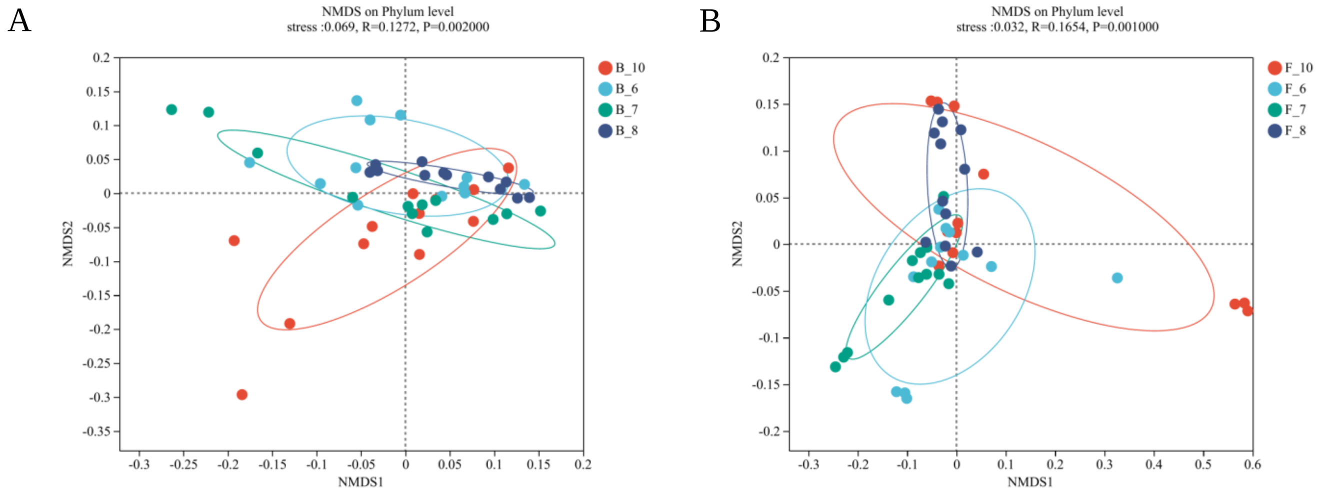

Table S1 Alpha diversity index statistics of bacterial grouped by cropping year

Estimators \ P value	Y_1 vs Y_2	Y_1 vs Y_3	Y_4 vs Y_1	Y_2 vs Y_3	Y_2 vs Y_4	Y_3 vs Y_4
ace	≥ 0.1	≥ 0.1	< 0.05	≥ 0.1	< 0.05	< 0.1
chao	≥ 0.1	≥ 0.1	< 0.001	≥ 0.1	< 0.001	< 0.05
shannon	≥ 0.1	≥ 0.1	< 0.05	≥ 0.1	< 0.01	< 0.05
simpson	≥ 0.1	≥ 0.1	< 0.1	≥ 0.1	< 0.1	< 0.1
sobs	≥ 0.1	≥ 0.1	< 0.01	≥ 0.1	< 0.001	< 0.05

Table S2 Alpha diversity index statistics of bacterial grouped by sample month

Estimators \ P value	B_10 vs B_6	B_10 vs B_7	B_8 vs B_10	B_6 vs B_7	B_6 vs B_8	B_7 vs B_8
ace	≥ 0.1	≥ 0.1	≥ 0.1	≥ 0.1	≥ 0.1	≥ 0.1
chao	≥ 0.1	≥ 0.1	≥ 0.1	≥ 0.1	≥ 0.1	≥ 0.1
shannon	< 0.1	≥ 0.1	< 0.05	≥ 0.1	≥ 0.1	≥ 0.1
simpson	< 0.1	≥ 0.1	< 0.1	≥ 0.1	≥ 0.1	≥ 0.1
sobs	≥ 0.1	< 0.1	≥ 0.1	≥ 0.1	≥ 0.1	< 0.05

Table S3 Alpha diversity index statistics of fungal grouped by cropping year

Estimators \ P value	Y_1 vs Y_2	Y_1 vs Y_3	Y_4 vs Y_1	Y_2 vs Y_3	Y_2 vs Y_4	Y_3 vs Y_4
ace	≥ 0.1	< 0.01	< 0.001	≥ 0.1	< 0.01	≥ 0.1
chao	≥ 0.1	< 0.05	< 0.001	≥ 0.1	< 0.001	< 0.01
shannon	≥ 0.1	≥ 0.1	< 0.001	≥ 0.1	< 0.001	< 0.001
simpson	≥ 0.1	≥ 0.1	< 0.05	≥ 0.1	< 0.05	< 0.01
sobs	≥ 0.1	≥ 0.1	< 0.001	≥ 0.1	< 0.001	< 0.001

Table S4 Alpha diversity index statistics of fungal grouped by sample month

Estimators \ P value	F_10 vs F_6	F_10 vs F_7	F_8 vs F_10	F_6 vs F_7	F_6 vs F_8	F_7 vs F_8
ace	< 0.05	≥ 0.1	< 0.05	≥ 0.1	≥ 0.1	≥ 0.1
chao	< 0.05	≥ 0.1	< 0.05	≥ 0.1	≥ 0.1	≥ 0.1
shannon	≥ 0.1	≥ 0.1	≥ 0.1	≥ 0.1	≥ 0.1	≥ 0.1
simpson	≥ 0.1	≥ 0.1	≥ 0.1	≥ 0.1	≥ 0.1	≥ 0.1
sobs	< 0.05	≥ 0.1	< 0.1	≥ 0.1	≥ 0.1	≥ 0.1
